# A Pediatric Patient With Autism Spectrum Disorder and Comorbid Compulsive Behaviors Treated With Robot-Assisted Relaxation: A Case Report

**DOI:** 10.7759/cureus.22409

**Published:** 2022-02-20

**Authors:** Vasiliki Aliki Nikopoulou, Vasiliki Holeva, Paraskevi Tatsiopoulou, Vassilis G Kaburlasos, Athanasios E Evangeliou

**Affiliations:** 1 Clinical Psychology Department, Papageorgiou General Hospital, Thessaloniki, GRC; 2 1st Psychiatric Clinic, Papageorgiou General Hospital, Thessaloniki, GRC; 3 HUman-MAchines INteraction Laboratory (HUMAIN-Lab), International Hellenic University, Kavala, GRC; 4 4th Department of Pediatrics, Division of Child Neurology and Metabolism, Papageorgiou General Hospital, Thessaloniki, GRC

**Keywords:** compulsions, case study, robot-assisted relaxation intervention, ocd, autism spectrum disorder

## Abstract

The nature of autism spectrum disorders (ASDs) presents significant challenges, especially with regard to comorbidities and drug treatments. Parents and caregivers are often hesitant towards psychotropic medications, mainly due to the fear of side effects. Problems arise when comorbid symptomatology reaches extreme levels, leading to functional decline in the patients. We discuss the case of a 13-year-old girl diagnosed with autism disorder who presented with a long history of social, interpersonal, and academic challenges. The patient was hospitalized with the complaint of a persistent, non-painful, and unpleasant sensation in the perineal area that eventually resulted in repetitive and compulsive behaviors. Robot-enhanced relaxation training was introduced to support the patient since she declined to undergo any form of talk therapy. The aim of the intervention was to prevent the irritation from escalating and promote self-regulation skills. The results, based on parent reporting, indicated that the patient acquired relaxation skills, experienced some positive effects on emotional regulation, and showed a decrease in the duration of her disruptive behaviors upon completing the relaxation training. This case report provides evidence that robot-assisted relaxation training may be effective in dealing with ASD-related behavioral disturbances and comorbid anxiety.

## Introduction

Autism spectrum disorders (ASDs) refer to a group of complex neurodevelopmental disorders that can cause significant social, communication, and behavioral difficulties. ASD is associated with a high psychiatric comorbidity burden [e.g., anxiety disorders, mood disorders, schizophrenia spectrum, impulse-control, conduct disorder, and attention deficit hyperactivity disorder (ADHD)] and heterogeneity in clinical symptoms [1].

The identification of obsessive-compulsive disorder (OCD) in the context of ASD can be challenging, due to their shared and overlapping features such as compulsive and ritualistic patterns of behaviors [2]. A characteristic feature of OCD is its ego-dystonic character, where the thoughts and compulsions experienced cause discomfort and patients try to resist them, in contrast with autistic rituals that can be rewarding and patients may even react aggressively if they are prevented from engaging in these activities [3]. Even so, it may be difficult to differentiate OCD symptoms from autistic ritualistic behaviors in cases of comorbid diagnosis [4].

Pharmacological treatment options including antipsychotic agents and stimulant medications are often used to address the symptoms of ASD and co-occurring conditions [5] and are considered effective in alleviating stereotypies, irritability, and obsessions [6], yet a risk-benefit analysis of pharmacotherapy should be performed in each case [7]. On top of that, caregivers appear to have a hesitant attitude towards psychotropic medications [8].

A range of non-pharmacological interventions have also indicated positive therapeutic effects; however, evidence-based guidance for their use in the management of ASD symptomatology is still not conclusive [9]. There has been a growing interest in newer and innovative modalities for the treatment of psychiatric disorders. For example, digital health interventions are being increasingly utilized for the treatment of ADHD [10]. Similarly, robot-assisted therapy has drawn considerable attention with respect to providing therapy in children with ASD. Several studies have utilized robots to manage repetitive and stereotyped behaviors [11].

This case report aims to explore the implementation of a social robot-assisted relaxation intervention as an alternative to standard treatment options to regulate negative emotions and de-escalate disruptive behaviors in a hospital setting.

## Case presentation

Background information

This paper presents the case of a 13-year-old girl diagnosed with ASD [level 1; Childhood Autism Rating Scale, Second Edition (CARS2) raw score: 24.5] with borderline intellectual functioning [the Wechsler Intelligence Scale for Children, Fifth Edition (WISC-V): IQ of 73; classification: very low; range 68-80; weaknesses on fluid reasoning and processing speed]. She had been receiving intervention treatments since the age of two (i.e., speech and language therapy, occupational therapy, physical therapy, psychological care, sensory integration therapy, dietary intervention). She now demonstrated a range of developmental impairments covering several domains (i.e., social, interpersonal, and academic) as well as emotional dysregulation, sleep difficulties, gastrointestinal symptoms, and stool withholding. She had two siblings and no family history of autism.

Physical examination

She visited the outpatient pediatric clinic during the summer complaining of a persistent, non-painful, and unpleasant sensation in the perineal area. This symptom was interpreted as an abnormal reaction to a noxious sensation since no related medical or organic cause was identified after thorough pediatric, gynecologic, and surgical examinations. Medical instructions were given and a follow-up appointment was scheduled, but no relief was reported.

During the following months, the predominant problem, as reported by the family, was school refusal, which was aggravated by the patient's excessive worry and irritability related to the frequency, intensity, and duration of the perceived sensation, resulting in daily meltdowns. In an attempt to relieve this unpleasant sensation, she gradually developed an abnormal pattern of behavior, which she could not control despite her efforts; she was driven to perform distressing recurrent actions, which eventually led to skin irritation and pain in the perineal area. The episodes were long-lasting (over six hours) with considerable and unpredictable escalation and manifested as repetitive and compulsive behaviors including rubbing and obsessive cleansing of her anus and genitals and/or excessive application of ointments or lotions. She also presented with explosive reactions (screaming, shouting, crying, and kicking the floor).

Her behavior gradually became so extreme, causing a marked functional decline. The family struggled to safely manage the situation at home and strongly believed that the perceived sensation was related to an underlying medical condition; therefore, she was admitted to the hospital for further evaluation. During her hospitalization, she was unable to cooperate with the doctors for the medical examinations and was referred to the psychiatric clinic.

After a psychiatric evaluation, the consultant child psychiatrist concluded that the patient lacked the ability for insight and did not perceive her compulsive behavior as bizarre. The compulsions were presented as self-contained rituals as she experienced relief from the act itself. An oral solution of risperidone 0.5 mg/ml was prescribed (once nightly); however, the family refused to comply with the prescribed medication, mainly due to fear of side effects.

Psychological intervention

The healthcare team reviewed the case at the weekly meeting and the psychology team decided to introduce robot-enhanced relaxation training to support the patient during hospitalization since she refused to engage in any form of talk therapy.

The aforementioned training has been provided by the psychology team as a hospital-based service since 2019, in order to support children with ASD in the context of a trial protocol [12] utilizing the social robot NAO, with proven effectiveness [13]. The proposed robot-enhanced relaxation training comprises both relaxation and mindfulness techniques. The social robot gives step-by-step instructions according to the relaxation scripts and offers positive feedback, while the therapist only directs the process and ensures safety. More specifically, the intervention protocol involves a breathing scenario (three levels), a progressive muscle relaxation scenario (three levels), a body scan meditation scenario (two levels), and a guided imagery scenario (three levels). The robot interacts with the child with both verbal and nonverbal cues using several sensors, such as cameras, microphones, eye color change, and body movements.

Since the expected length of her stay was scheduled to exceed 10 days, a daily-session program was initiated. The application of the training was intensive and short-term; therefore, the last two scenarios were implemented only for difficulty level 1. The training also included a familiarization and a closing session. All sessions took place in the multi-sensory room of the clinic for approximately 30 minutes each.

**Figure 1:**
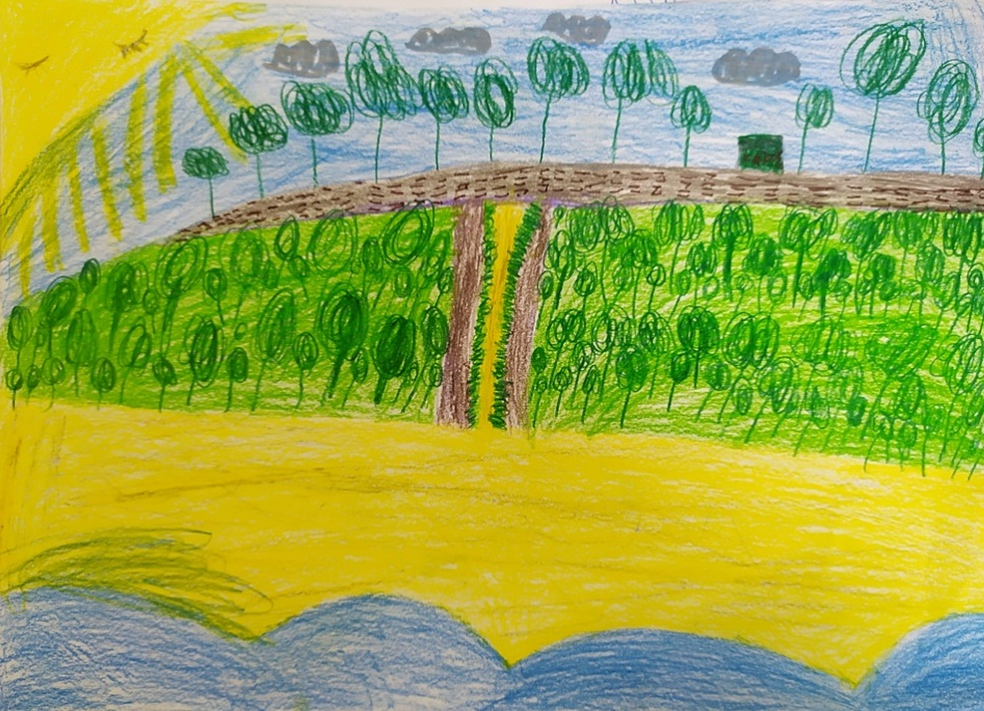
Drawing by the patient after the guided imagery scenario

The training intervention was intended to prevent her irritation from escalating, increase awareness, manage physiological arousal, and promote self-regulation skills. From the beginning, she seemed quite motivated to participate in the training and interact with the robot. She was very willing to meet NAO during the sessions and frequently talked about him to her parents and doctors. The repeated training routine encouraged her to practice the relaxation techniques more often during the day and create positive experiences in the hospital setting. Figure 1 presents the drawing she made based on the safe place imagery script in the last session.

**Figure 2:**
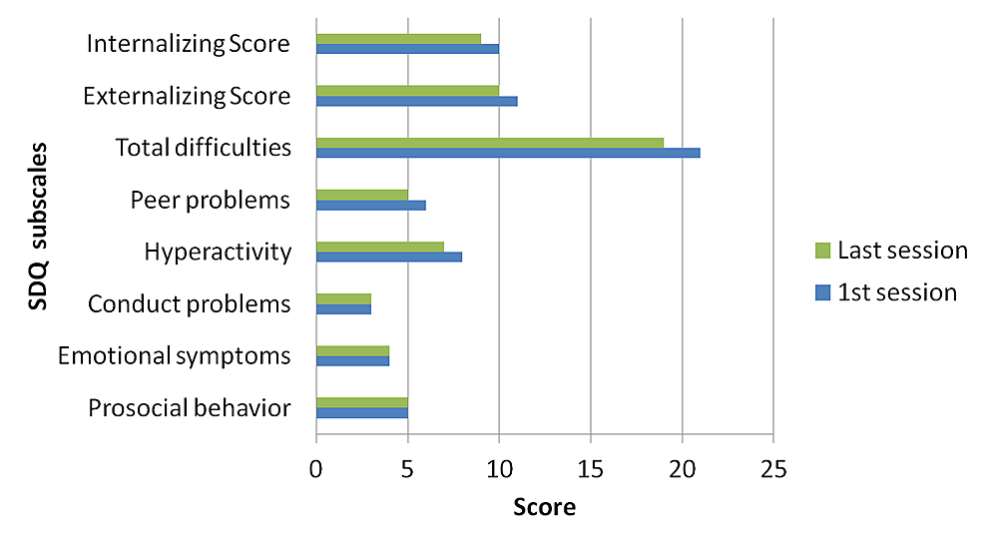
Mother’s ratings The graph is based on the Strengths and Difficulties Questionnaire (SDQ)

**Figure 3:**
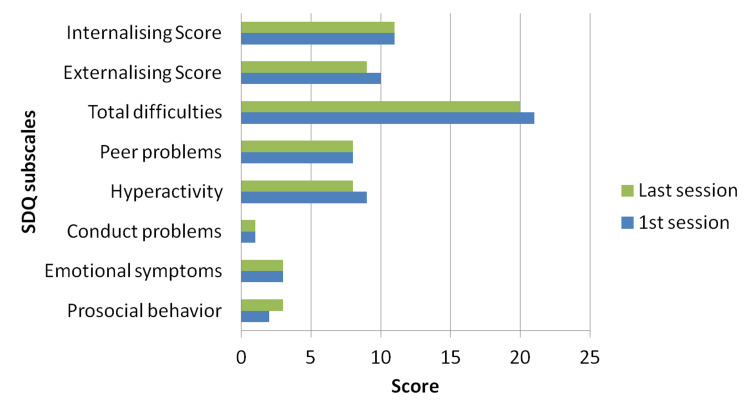
Father’s ratings The graph is based on the Strengths and Difficulties Questionnaire (SDQ)

The Strengths and Difficulties Questionnaire (SDQ) [14,15] was completed by the parents before and after the training. SDQ is a 25-item emotional and behavioral screening questionnaire assessing hyperactivity/inattention and conduct problems (i.e., externalizing score), emotional symptoms and peer problems (i.e., internalizing score), as well as positive prosocial behavior. Low levels of prosocial behavior and SDQ total scores of 17 and above are considered to be abnormal. According to the family’s rating, her difficulties pre- (mean total difficulties score: 21) and post-training (mean total difficulties score: 19.5) were within the abnormal range; however, a slight decrease was observed post-training (Figures 2, 3).

In addition, a symptom-tracking diary was filled out to detect potential mood and behavior alterations. The logs revealed a slight decline in the intensity and duration of her meltdowns, inability to sleep, and irritability. It is also worth noting that although during her hospital stay her parents insisted that she had an underlying medical condition, after her discharge, they seemed more receptive to the idea that the psychological component was the prevailing issue.

## Discussion

When patients suffer an exacerbation in OCD/anxiety symptoms or when a meltdown is in progress, the management can be difficult and there is no conclusive research that provides an effective strategy or a "gold standard" approach. Non-adherence to prescribed medications by patients/caregivers disturbs the procedures often used in hospitals to target stress and challenging behaviors.

This case study provides an insight into adopting the use of social robots as co-therapists or relaxation instructors in hospital and emergency care settings. The positive influence of the robot-enhanced relaxation training, as a complementary therapy, was addressed in our case by the parental reports and the healthcare team observations. The findings are in line with previous studies in terms of the reduction of the negative emotional intensity after a robot-enhanced cognitive-behavioral treatment [16], as well as the patient's heightened self‐monitoring, self-control, and positive emotional response after robot-based mindfulness training [17].

Another key observation in this case report was regarding the level of the adolescent’s engagement and motivation. It was obvious that she was more enthusiastic to interact with the social robot than with the therapist alone. Considering that a hospital setting is especially challenging for youths with ASD, the focus can be given to the effectiveness of more personalized and technology-based intervention options. Furthermore, robot-based intensive and short-term interventions can benefit from their "novelty effect", which elicits higher engagement with the robot and the task, especially during the initial sessions [18].

The use of social robots in healthcare contexts seems quite promising. Robots can promote a wide array of positive effects, including storing data, preventing isolation-related challenges, and facilitating edutainment activities in the pediatric ward [19]. The incorporation of interactive social robots as social mediators, instructors, or screening agents is a work that is still in progress and there is a great interest in integrating evidence-based treatment techniques. For instance, a recent publication proposes robot-based therapeutic scenarios for social anxiety disorders to inspire future empirical work [20]. Nonetheless, further research is needed to demonstrate the reliability and consistent efficacy of such intervention protocols. More investigations on patient and implementation characteristics would probably contribute to refining robot-based interventions further.

## Conclusions

This is the first case report on implementing relaxation training to an adolescent with ASD during hospitalization by utilizing a social robot. The focus was on providing a quick and effective alternative to augment conventional treatment and reduce psychological and behavioral disturbances. The outcomes were encouraging, yet only indicative. More empirical work is needed to further appraise the use of robots in healthcare settings.
